# Low-level viremia in people with HIV in Ethiopia is associated with subsequent lack of viral suppression and attrition from care

**DOI:** 10.1080/16549716.2025.2464342

**Published:** 2025-02-13

**Authors:** Ilili Jemal Abdulahi, Per Björkman, Alemseged Abdissa, Patrik Medstrand, Anton Reepalu, Olof Elvstam

**Affiliations:** a Department of Translational Medicine, Lund University, Malmö, Sweden; b Agenda 2030 Graduate School, Lund University, Lund, Malmö, Sweden; c Viral Disease Research Division, Armauer Hansen Research Institute, Addis Abeba, Ethiopia; d Department of Infectious Diseases, Skåne University Hospital, Sweden; e Department of Infectious Diseases, Växjö Central Hospital, Växjö, Sweden

**Keywords:** HIV, low-level viremia, virologic non-suppression, dolutegravir, Ethiopia

## Abstract

**Background:**

Low-level viremia during antiretroviral therapy (ART) has been associated with inferior outcomes, but knowledge on the impact of low-level viremia in the current era of dolutegravir-based ART in low-income countries is limited.

**Objective:**

To investigate whether low-level viremia predicts virologic non-suppression and attrition from care in people with HIV receiving ART in Ethiopia.

**Methods:**

We included people receiving ART at public health facilities in an urban area in central Ethiopia and categorized persons with ≥1 available viral load 2019–2020 as having either suppression (<150 copies/mL) or low-level viremia (151–1,000 copies/mL); people with >1,000 copies/mL were excluded. We used multivariable logistic regression adjusted for age, sex, ART regimen, type of health facility, and duration of ART to analyze the associations between viremia category and incidence of unsuppressed viral load (>1,000 copies/mL) and attrition from care (death or loss to follow-up) during 3 years of follow-up.

**Results:**

Among 12,165 participants, the median age was 44 years, 64.2% were female, and 89.1% received tenofovir/lamivudine/dolutegravir. Of the study population, 11,959 (98.3%) had suppression and 206 (1.7%) had low-level viremia. Over 3 years of follow-up, 2.2% of participants with suppression and 11.3% with low-level viremia had unsuppressed viral load. Low-level viremia was associated with both unsuppressed viremia (adjusted odds ratio [aOR], 3.7; 95% confidence interval [CI], 2.2–6.2) and attrition (aOR, 3.4; 95% CI, 1.7–6.6).

**Conclusion:**

Among Ethiopian people with HIV receiving ART, low-level viremia predicted subsequent virologic non-suppression and attrition from care, supporting current recommendations for heightened attention to low-level viremia in ART recipients.

## Paper Context

- **Main findings**: Among people with HIV receiving antiretroviral therapy in Ethiopia (89.1% receiving tenofovir/lamivudine/dolutegravir), low-level viremia (HIV RNA 151–1000 copies/mL) was associated with higher odds of future unsuppressed viremia (HIV RNA > 1000 copies/mL) and attrition from care.
- **Added knowledge**: The previously known association between low-level viremia and worse treatment outcome among people with HIV is relevant even for those on dolutegravir-based regimens – a newer treatment with higher antiviral efficacy and resistance barrier.
- **Global health impact for policy and action**: Increased attention to low-level viremia during antiretroviral therapy is warranted in resource-limited settings, and future research should explore strategies to manage low-level viremia in such circumstances.

## Background

In most people with HIV (PWH) receiving antiretroviral therapy (ART), viral replication is suppressed, resulting in plasma viral load (VL) below the limit of quantification. Viral suppression leads to reduced HIV-related morbidity and mortality, while also interrupting sexual transmission [1]. Furthermore, effective suppression of viral replication prevents emergence of drug resistance mutations [1,2] and subsequent virologic failure. Whereas VL > 50–200 copies/mL is used to define virologic failure in high-income countries [3,4], the World Health Organization guidelines, which are used in most low-income countries, recommend repeated measurements of VL > 1,000 copies/mL as a definition of virologic failure [1]. In a proportion of persons receiving ART, HIV RNA is detected in plasma at levels below the threshold for virologic failure; this phenomenon is commonly referred to as low-level viremia (LLV) [1,5].

LLV is more common in people starting ART at advanced disease stage [6] and is considered to reflect viral release from the latent reservoir [7]. However, LLV can also be caused by ongoing HIV replication due to suboptimal ART. The varying criteria used to define LLV make it difficult to compare reported prevalences of this phenomenon during ART [8–13]. Several studies, performed in different settings, have found LLV to be associated with subsequent virologic failure [12–15]; in addition, LLV has been linked to increased mortality in some studies [16–18].

Most previous research on LLV originates from Europe and North America, while there is limited data from sub-Saharan Africa, where the majority of PWH live. Furthermore, most previous studies mainly include people receiving non-nucleoside reverse transcriptase inhibitor (NNRTI)-based or protease inhibitor (PI)-based ART. With the recent rollout of tenofovir/lamivudine/dolutegravir (TLD) as first-line regimen globally, there is a need for data on consequences of LLV among TLD recipients in low-income countries. In this study, we report the prevalence of LLV in PWH receiving ART in Ethiopian public care after the introduction of dolutegravir-based ART, and associations between LLV and unfavorable virologic outcome and attrition from care.

## Methods

### Study settings and participants

In this retrospective study, we used routinely collected program data from eight clinics providing ART free of charge in public health facilities (two hospitals and six health centers) in four urban areas in the central Oromia region, Ethiopia (Adama, Mojo, Olenchity, and Bishoftu). These areas are located along a major transport highway and have higher HIV prevalence than most other parts of Ethiopia (2.4–3.4%, compared to the national average of 0.96% [19]).

During the study period, Ethiopian guidelines recommended routine VL testing at 6 and 12 months after initiating ART and then annually, except for people with VL results >1,000 copies/mL, for whom repeated VL testing was recommended after 3 months of enhanced adherence counselling. For this study, all PWH receiving ART at any of the study sites with at least one documented VL result during June 2019–June 2020 in an electronic database were included. People with VL > 1,000 copies/mL during the inclusion period were excluded from our main analysis since they met the virologic outcome criterion non-suppression. All ART sites in the uptake area send samples for VL testing to the Adama Public Health Research and Referral Laboratory. Abbott RealTime HIV-1 assay m2000 Systems was used for VL testing, with a lower quantification limit of 150 copies/mL (since a 0.2 mL sample volume procedure was used at this laboratory).

At ART clinic visits, patient data (including sociodemographic, clinical, laboratory test results and follow-up visit information) are routinely entered into electronic ART databases, which were used as data sources for this study. Data on ART adherence, CD4 counts, and World Health Organization stage were only available for manual data scrutiny and were not included for analysis. The following variables were available for automatic data management and included in the analysis: age, sex, duration on ART, regimen type, viral load, and type of health facility. Data clerks based at the selected ART clinics generated data files with selected study variables in Microsoft Excel format from electronic database. ART codes (unique for each individual) were used to link participants’ VL data with other variables.

Ethical clearance was granted from the Armauer Hansen Research Institute review board, Addis Ababa, Ethiopia (Protocol number P039/23).

### Categorization of viremia

Persons with VLs of <150 copies/mL were categorized as having virologic suppression, while those with VL in the range 151–1,000 copies/mL were categorized as having LLV. We used the first VL result available during the inclusion period to categorize participants. Participants could only belong to one viremia category.

### Outcome definitions

Participants were assessed for virologic outcomes and attrition from care during a follow-up period of 3 years after viremia categorization as primary and secondary outcome, respectively (last date of follow-up, June 2023). Persons with at least one follow-up VL during this period were evaluated for virologic outcome by the highest VL detected during the 3-year period (the outcome unsuppressed VL defined as VL > 1,000 copies/mL). Separately, we determined the number of participants with persistent LLV during follow-up among those with LLV at inclusion. Participants whose highest HIV RNA levels were in the LLV range during follow-up VL were classified as persistent LLV (≥ one follow-up VL result required over 3-year follow-up).

The outcome attrition from care was defined as loss to follow-up (LTFU; defined as absence from care for at least 30 days from the last scheduled appointment date and without re-engagement to care at the study sites during the follow-up period) or documented death. Participants with documented transfer of care during the study period were excluded from the attrition from care analysis, as well as those with missing information on follow-up status.

### Statistical analysis

Median with interquartile range (IQR) was used to summarize continuous variables, and frequency and percentage were computed for categorical variables. We used univariable and multivariable logistic regression to analyze the associations between viremia category and treatment outcomes. In the model for virologic outcome, we adjusted for age at inclusion, sex, duration of ART before inclusion, type of health facility and regimen type. The same variables were included in the attrition model, except regimen type, which was excluded due to low number of occurrences per outcome category (fewer than ten). We checked the linearity between the continuous predictors and the logits using Box-Tidwell transformation. We also performed a sensitivity analysis only including those receiving TLD for the virologic outcome analysis.

## Results

### Participants

**Figure 1.**
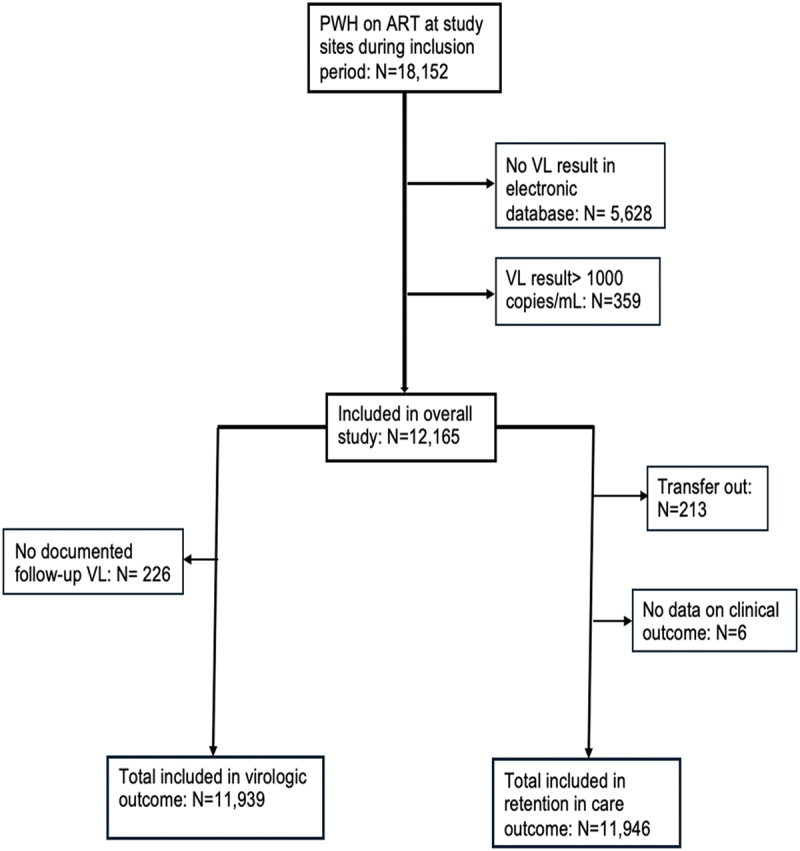
Flow chart of study inclusion and exclusion. Abbreviations: PWH, People with HIV; ART, antiretroviral therapy; VL, Viral load.

In total 18,152 PWH were receiving ART at the study sites during the inclusion period, among whom 12,524 (69.0%) had a documented VL result (Figure 1). Of those, 359 (2.9%) individuals had VL > 1,000 copies/mL and were excluded. Among the 12,165 included participants with VL ≤ 1,000 copies/mL 11,959 (98.3%) had virologic suppression and 206 (1.7%) had LLV.

Among included participants, the median age was 44 years (IQR, 37–51) and 64.2% were female. The median duration of ART before inclusion was 8.2 years (IQR, 4.7–11.2). At inclusion, 153 (74.3%) of those with LLV and 10,687 (89.4%) of those with virologic suppression received TLD. Overall, 36.8% of participants received care at health centers and 63.2% at hospitals. The proportion who received care at health centers was higher among people with LLV (52.4%, compared with 36.6% among those with viral suppression) (Table 1).

**Table 1.** Characteristics of study participants by viremia category at study inclusion, *N* = 12,165.

		Viral suppression (<150 copies/mL)	LLV (151–1000 copies/mL)
Age (years)		44.0 (37.0–51.0)	40.0 (25.8–46.3)
Time since ART initiation to study inclusion (years)		8.2 (4.8–11.2)	7.7 (4.3–10.9)
Age			
	<15 years	192 (1.6%)	15 (7.3%)
	≥15 years	11,767 (98.4%)	191 (92.7%)
Sex			
	Female	7688 (64.3%)	125 (60.7%)
	Male	4271 (35.7%)	81 (39.3%)
ART regimen*			
	1^st^ line	10,848 (90.7%)	162 (78.6%)
	2^nd^ line	720 (6.0%)	27 (13.1%)
	3^rd^ line	12 (0.1%)	0 (0.0%)
	Missing data	379 (3.2%)	17 (8.3%)
Type of health facility			
	Health center	4372 (36.6%)	108 (52.4%)
	Hospital	7587 (63.4%)	98 (47.6%)
Number of follow-up VL tests			
	0	214 (1.8%)	12 (5.8%)
	1	520 (4.3%)	11 (5.3%)
	2	3303 (27.6%)	52 (25.2%)
	3	7922 (66.2%)	131 (63.6%)

Results are number (%) except for age and time since ART initiation to study inclusion which are median with interquartile range.

*Preferred first-line regimen in Ethiopia at time of this study was dolutegravir-based ART, while second-line (atazanavir or lopinavir) and third-line (darunavir) regimens were based on boosted protease inhibitors unless contradicted. Tenofovir/lamivudine/dolutegravir was the most common regimen among participants on first-line ART (98.5%) while tenofovir/lamivudine/atazanavir (49.4%) and zidovudine/lamivudine/atazanavir (31.6%) were the two most common second line regimens. The most common third-line regimens were darunavir/dolutegravir/zidovudine/lamivudine (41.7%) and darunavir/dolutegravir/tenofovir/lamivudine (33.3%).

Abbreviations: ART, antiretroviral therapy; LLV, low-level viremia; VL, Viral load.

Among people with LLV, the median VL at study inclusion was 390 copies/mL (IQR 248–631). Twenty-five individuals (12.1%) had LLV in the range 151–200 copies/mL, 111 (53.9%) in the range 201–500 copies/mL, and 70 (34.0%) in the range 501–1,000 copies/mL.

### Association between viremia category and virologic outcome

**Figure 2.**
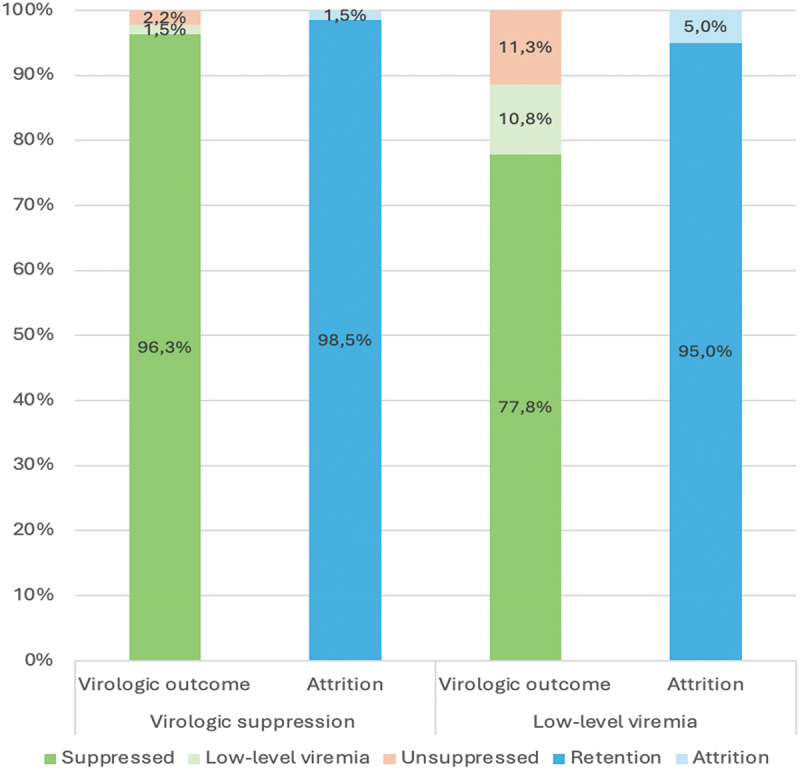
Follow-up viral load and attrition status for virally suppressed and low-level viremia group. Participants were grouped by viremia at inclusion into virologic suppression (VL <150 copies/mL) and low-level viremia (VL 151–1000 copies/mL) and followed for 3 years. Follow-up viremia category was defined as the highest VL during follow-up and grouped as suppressed (VL <150 copies/mL), low-level viremia (VL 151–1000 copies/mL) or unsuppressed (VL > 1000 copies/mL). Attrition from care was defined as loss to follow-up or death. VL, viral load.

During follow-up, a median of 3 VL results (IQR 2–3) were available from each study participant. Most persons with virologic suppression (66.2%), as well as with LLV (63.6%), had 3 follow-up VL results over 3 years, while 226 (1.9%) (12 LLV and 214 viral suppression) participants had no documented VL results and were hence excluded from the virologic outcome analysis (Table 1). From the 12 LLV participants with no follow-up VL, 1 died and 4 were LTFU. Similarly, from 214 participants in suppression group with no follow-up VL, 23 died and 50 were LTFU. Among a total of 11,939 participants included in the virologic outcome analysis, 286 (2.4%) had unsuppressed VL (>1000 copies/mL) during follow-up. Having unsuppressed VL was more common among people with LLV compared with virologic suppression (11.3% vs 2.2%). Of the 194 participants with LLV during the inclusion period and who had a follow-up VL, 21 (10.8%) had persistent LLV during follow-up (Figure 2).

In univariable analysis, participants with LLV had statistically significantly higher odds of unsuppressed VL (>1000 copies/mL). The association remained statistically significant after adjustment for age, sex, type of health facility, and duration of ART (adjusted odds ratio [aOR], 3.73; 95% confidence interval [CI], 2.24–6.20) (Table 2). This association remained unchanged in a sensitivity analysis restricted to participants receiving TLD at inclusion (aOR, 3.88; 95% CI, 2.03–7.40).

**Table 2.** Logistic regression models for 3-year risk of unsuppressed viral load (VL > 1,000 copies/mL) and attrition from care depending on viremia category.

		Yes, n (%)	No, n (%)	Unadjusted OR*	Adjusted OR*
Unsuppressed VL > 1000 copies/mL** (*n* = 11,939)					
Viremia category					
	Suppression	264 (2.2%)	11481 (97.8%)	1 (Ref.)	1 (Ref.)
	LLV	22 (11.3%)	172 (88.7%)	5.6 (3.5–8.8)	3.7 (2.2–6.2)
Attrition from care*** (*n* = 11,946)					
Viremia category					
	Suppression	173 (1.5%)	11574 (98.5%)	1 (Ref.)	1 (Ref.)
	LLV	10 (5.0%)	189 (95.0%)	3.5 (1.8–6.8)	3.4 (1.7–6.6)

*All results are odds ratios with 95% confidence intervals.

**Virologic outcome models were adjusted for age, sex, ART regimen, type of health facility, and duration of ART before study inclusion. Unsuppressed VL was defined as any VL > 1000 copies/mL during 3 years of follow-up.

***Attrition models were adjusted for age, sex, type of health facility, and duration of ART before study inclusion. Attrition of care included those with documented death during follow-up and those lost to follow-up (defined as absence from care for ≥30 days from last scheduled appointment and without re-engagement to care at the study sites during the follow-up period).

Abbreviations: LLV, low-level viremia; Ref., reference category; VL, viral load; OR, odd ratio.

### Association between viremia category and attrition from care

A total of 11,946 participants were included in this analysis (11,747 [98.3%] with virologic suppression and 199 [1.7%] with LLV) (Figure 1). At the end of 3 years of follow-up 11,763 (98.5%) participants were alive on ART, while 152 and 31 individuals were LTFU and dead, respectively. Among persons with virologic suppression, attrition rate was lower (1.5%; 30 deaths and 143 LTFU) than among people with LLV (5.0%; 1 death and 9 LTFU). People with LLV had statistically significantly higher odds of attrition compared with persons with virologic suppression (aOR, 3.35; 95% CI, 1.71–6.56) (Table 2).

## Discussion

In this register study of PWH receiving ART at Ethiopian public health facilities, we found that LLV predicted subsequent lack of viral suppression, as well as attrition from care.

During the last 20 years, a vast increase in access to ART throughout sub-Saharan Africa has been made possible through simplified provision of care, standardized regimens, task-shifting, and minimized laboratory monitoring. Universal VL monitoring has been recommended since 2016, in response to increasing resistance to NNRTI, the previous first-line drugs. Furthermore, first-line ART recommendations were changed in 2018 to dolutegravir-based regimens (mainly TLD), with higher barriers to resistance. In Ethiopia, DTG roll-out started early 2019 [20]. Although WHO guidelines still use 1,000 copies/mL as the threshold for virologic failure, the updated treatment monitoring algorithm from 2021 recommends enhanced adherence counselling followed by repeated VL testing for people with LLV [1]. This recommendation was based on observational data indicating higher risk of virologic failure among people with LLV [13,14]. However, these studies were performed before the rollout of TLD, and data on the impact of LLV in people receiving dolutegravir-based ART are limited. Our study, with a majority of participants receiving TLD, shows that LLV is associated with inferior treatment outcomes, thus providing evidence to support the recommendation of increased attention for LLV in ART recipients.

First, we observed a higher risk of future lack of viral suppression among people with LLV. An association between LLV and inferior virologic outcome has previously been reported from different settings, in particular when the LLV definition included people with VL in the range 200–999 copies/mL [8,12–15,21–24]. Several of these studies have been performed in sub-Saharan Africa. A multi-center South African cohort study observed higher risk of virologic failure for people with LLV (defined as 51–999 copies/mL) compared with VL < 50 copies/mL [13]. Another observational cohort study (with participants from Uganda, Kenya, Tanzania, and Nigeria) reported double risk of virologic failure for persistent LLV ≥ 200 copies/mL compared with undetectable VL [14]. In addition, a randomized controlled trial from Lesotho found that participants with LLV (100–999 copies/mL) receiving first-line NNRTI-based ART switching to second-line protease-inhibitor-based ART had higher probability of viral suppression compared with those kept on first-line treatment [25]. These three studies have included people receiving first-line NNRTI-based regimens. Since dolutegravir-based regimens have higher antiviral efficacy and resistance barrier, the occurrence and impact of LLV could be lower among people receiving TLD. We are aware of one recent study on virologic outcomes in TLD recipients; Kohler *et al*. observed lower occurrence of LLV among dolutegravir recipients compared with those receiving NNRTI in Lesotho, but both low- (50–199 copies/mL) and high-range LLV (200–999 copies/mL) was associated with subsequent virologic failure irrespective of core agent [26]. Our study corroborates these findings, implying that consideration of LLV is relevant also for TLD.

Second, besides virologic outcome, we assessed associations between LLV and attrition from care and found higher odds of attrition (defined as death and loss to follow-up) for participants with LLV compared to those with virologic suppression. An association between LLV and mortality has previously been reported in some studies [16–18]. However, this finding is not universal [8,9], and the potential underlying mechanisms are unclear. To our knowledge, the associations between LLV and attrition from care have not previously been studied in sub-Saharan Africa. Due to the high rate of loss to follow-up (without further information on outcome, constituting 83% of cases of attrition from care) we were unable to determine whether LLV was associated with mortality. However, a considerable fraction of study participants categorized as LTFU in our setting might represent unrecognized fatalities. A study from Ethiopia that investigated outcomes for PWH lost from care after ART initiation found that 22.9% of those who could be traced had died [27]. In this setting, loss to follow-up can therefore be considered a clinically relevant outcome.

Third, we found a relatively low prevalence of LLV compared with previous studies. Interestingly, we noted that only 69% of ART recipients eligible for inclusion had an available VL measurement documented in the electronic data base during the 1-year inclusion period. This indicates that VL coverage in the uptake area may be lower than stated in official reports [28], although the coverage likely would have been higher if we had analyzed a slightly wider time window (if the VL frequency was just over 12 months in some cases). Unfortunately, data on the 31% with no documented VL during the study period were not available, which is a limitation of our work. Different definitions of LLV exist in the literature; apart from the amplitude of viremia, there are also differences in number and frequency of VL results available for classification. In most studies from high-income settings, the definition of LLV is based on at least two consecutive VL results obtained within defined time periods [8,11,29]. However, several studies have used single VL results to define LLV in settings where multiple routine VL results are not available [12,13,26], which represent settings where most PWH in the world receive care. The prevalence of 1.7% LLV among persons with VL ≤ 1,000 copies/mL is lower than in most previous studies [8–10,12–14]. This difference could be explained by lower risk of LLV during dolutegravir-based first-line ART compared with NNRTI-based regimens [26], as well as by the lower limit of quantification of 150 copies/mL used for routine virologic monitoring in our study population, which is higher than that used in most previous studies [8,12–14,24].

A recent randomized study from Uganda showed that enhanced adherence counseling led to nearly double chance of achieving VL < 50 copies/mL in people with LLV, compared with participants in the non-intervention arm [30]. Apart from supporting the effectiveness of adherence counseling for the management of LLV, this finding also implies that ongoing viral replication (usually due to insufficient adherence) may be a common cause of LLV in sub-Saharan African settings. We did not have data on ART adherence or adherence counseling. Further investigations of LLV in connection to TLD in sub-Saharan Africa are warranted, for example, to understand the mechanisms of LLV, the role of LLV in the emergence of dolutegravir resistance, and in which cases switch to alternative ART regimens might be beneficial.

Our study has certain limitations. First, since we defined LLV based on single VL results, we were unable to separate LLV and viral blips. However, such transient episodes of viremia have also been found to be associated with future lack of viral suppression [15]. Indeed, our study shows that even a single VL result in the LLV range may be used as a predictor of worse treatment outcome. Second, we included a restricted number of variables for our analyses due to lacking data for several characteristics that might have been of interest (for example, treatment adherence, World Health Organization stage, CD4 counts, and comorbidities). Third, we consider our findings to be representative of urban and semi-urban areas in Ethiopia, but since health facilities in rural areas were not included, our results may not be generalizable to the whole country. Fourth, the assay used for VL testing had a detection limit of 150 copies/mL, and we were therefore unable to study LLV at lower amplitudes. We had few participants with a VL of 151–200 copies/ml and were unable to perform a sub-analysis of LLV < 200 copies/mL. VL testing was done at the same laboratory using the same type of VL assay throughout the study period, which is a strength of our study, considering inter-assay variations in the LLV range [31]. Another strength of our study is the large population of PWH receiving routine HIV care at public health facilities in Ethiopia.

In conclusion, our study shows that LLV is associated with inferior virologic outcomes and attrition from care in PWH in Ethiopia, mainly receiving dolutegravir-based ART. Our findings suggest that VL in the LLV range may be used to predict adverse ART outcomes and provide evidence to support the World Health Organization guidelines for heightened attention to PWH with LLV during dolutegravir-based ART.

## Data Availability

The patient level data cannot be shared publicly but Oromia Regional Health Bureau can grant access upon request (Email: info@orhb.gov.et) for those who meet criteria for access to confidential data. The data are fully owned by Oromia Regional Health Bureau and respective health facilities.
